# Deep Neck Infection: A Case of Retropharyngeal Abscess

**DOI:** 10.7759/cureus.48293

**Published:** 2023-11-05

**Authors:** Joana Martins, Ana Lucas

**Affiliations:** 1 General Physician, Unidade de Saúde Familiar (USF) São Vicente, Porto, PRT; 2 General Physician, Unidade de Saúde Familiar (USF) Renascer, Porto, PRT

**Keywords:** pharyngitis, otorhinolaryngologic diseases, bacterial infections, respiratory tract infections, retropharyngeal abscess

## Abstract

The incidence of retropharyngeal abscesses has been decreasing since the introduction of antibiotic therapy, and it is currently a rare diagnosis in adults, although there are some recent cases in the literature. Given its seriousness, if not treated promptly, the infection can progress rapidly and its complications can be fatal, making it a serious health problem.

A 79-year-old woman presented at her primary care center with complaints of persistent odynophagia for about two weeks and rapidly progressive dysphagia in five days, initially for solids and later for solids and liquids. On observation, she had difficulty swallowing saliva and presented a painful mass in the bilateral anterior submental and anterior cervical region. Due to the rapid progression of symptoms and the findings of the physical examination, the patient was referred to the emergency department for a suspected abscess or cervical mass. In the emergency department, a cervical CT scan was performed, which revealed a retropharyngeal abscess measuring approximately 7 x 6 x 4 cm, involving the right carotid artery and internal jugular vein, with compression of the internal jugular vein. The patient was admitted to the otorhinolaryngology department, where intravenous antibiotic therapy with third-generation cephalosporin and clindamycin was initiated. She underwent exploration in the operating room to determine the cause of the abscess and transoral drainage of the already spontaneously draining abscess. After completing antibiotic therapy, a follow-up CT scan showed complete resolution of the abscess without suggestive masses of neoplasm or foreign bodies, therefore, the cause of the abscess has not been identified.

The most frequent cause of retropharyngeal abscess in adults is dental septic foci and another commonly described cause is the ingestion of foreign bodies such as fish bones or chicken bones. Early diagnosis of this condition is crucial, as delays in treatment initiation can lead to the progression of infection into the deep cervical spaces, resulting in serious complications such as mediastinitis, pericarditis, jugular vein thrombosis, sepsis, laryngeal edema, conditions with a high degree of morbidity and mortality. Therefore, it is important for any doctor to be aware of warning signs and symptoms in patients who present such symptoms, especially primary care doctors, who are the first gateway to health services and to whom patients often turn first. This case report shows the importance of suspicion and subsequent referral for timely diagnosis and treatment.

## Introduction

Deep neck infections are sited in the potential spaces and fascial planes of the neck within the limits of the deep layer of the cervical fascia, the retropharyngeal abscess being one of the most common [1]. A retropharyngeal abscess is an infection located in a virtual space between the pharynx and the cervical vertebrae that extends into the mediastinum [1]. The incidence of retropharyngeal abscesses has been decreasing since the introduction of antibiotic therapy, and it is currently an unusual diagnosis in adults, although there are some recent cases in the literature [2-4]. Given its seriousness, if not treated promptly, the infection can progress rapidly and its complications can be fatal, making it a serious health problem. The main aim of this paper is to present a clinical case of retropharyngeal abscess of unidentified etiology, focusing on its suspicion in the context of primary healthcare.

## Case presentation

A 79-year-old woman, married, living with her husband, retired, totally independent with a personal history of hypertension and dyslipidemia diagnosed 10 years ago and controlled, overweight, and heart failure with preserved ejection fraction diagnosed one year ago. Her surgical history included cataract correction surgery three years ago, vascular surgery for varicose veins six years ago, and complete excision of basal cell carcinoma in the nose eight years ago. Her regular medication includes lisinopril 20mg + amlodipine 5mg at breakfast, furosemide 40mg at lunch, and simvastatin 20mg at dinner. Informed consent was obtained to write this case report.

She presented to her primary care center with complaints of odynophagia for about one week and myalgia, without fever or other associated symptoms. On physical examination, she was afebrile, with no changes in auscultation, and had hyperemia oropharynx without uvula deviation, tonsillar hypertrophy, or evident exudates. She was diagnosed with an acute upper respiratory infection of viral origin and was prescribed symptomatic treatment. 

She returned after four days to the primary care center due to persistent odynophagia and rapidly progressive dysphagia, initially for solids and subsequently for both solids and liquids, having difficulty taking her regular medication. On observation, she had difficulty swallowing saliva. She also reported febrile symptoms such as chills, sweating, and asthenia, without recording her temperature at home. No dysphonia was reported, and she denied dyspnea, a history of choking on a meal, or other complaints. On physical examination, she was conscious, oriented, and cooperative, hemodynamically stable without any signs of respiratory distress. Her axillary temperature was 37.2ºC. She presented a painful mass in the bilateral anterior submental and anterior cervical region, making oral cavity examination difficult. Due to the rapidly evolving symptoms and the physical examination findings, the patient was referred to the emergency department for a suspected cervical abscess or mass.

**Figure 1:**
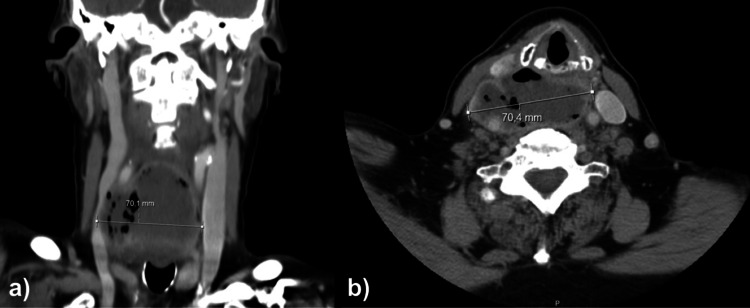
a) Image of the abscess on cervical computed tomography in coronal section. b) Image of the abscess on cervical computed tomography in cross-sectional.

In the emergency department, a cervical computed tomography (CT) with contrast scan was performed, revealing a large fluid collection containing gas bubbles and thickened walls, located posteriorly to the larynx without a clear cleavage plane with the esophagus, measuring approximately 7 x 6 x 4 cm (Figure 1). The appearance was difficult to interpret, as it could represent a collection secondary to esophageal perforation or esophageal neoplasm. Further evaluation by endoscopy was recommended. The mentioned collection involved the right carotid artery and internal jugular vein, with compression of the internal jugular vein.

The patient was admitted to the otorhinolaryngology department with a diagnosis of retropharyngeal abscess and intravenous antibiotic therapy was initiated with ceftriaxone 1000mg 12/12h and clindamycin 600mg 8/8h during 14 days. She underwent exploration in the operating room for the cause of the abscess and transoral drainage of the already spontaneously draining abscess. After completing antibiotic therapy, a follow-up CT scan showed complete resolution of the abscess without suggestive masses of neoplasm or foreign bodies. The microbiological results of the exudate were polymicrobial with Enterobacter cloacae, Staphylococcus epidermitis*,* and gram-positive aerobic bacilli sensitive to the administered antibiotic. The patient was discharged after 14 days of hospitalization. 

## Discussion

Retropharyngeal abscesses in adults are unusual, with the most frequent causes being dental septic foci and ingestion of foreign bodies, such as fish or chicken bones [2-5]. Less common causes include trauma, salivary gland infections, and intravenous drug use or administration of intravenous medications into the major veins in the neck [3,4]. However, in about 20% of cases, the etiology is not identified [2,3]. In this clinical case, no foreign body was found, and there was no history of choking on a meal or drug or medication intravenous. It's noteworthy that the assessment and description of dental conditions were lacking, which could have been a potential cause for the abscess; however, the patient did not present any complaints.

Regarding the clinical presentation, odynophagia (painful swallowing) is the most common, frequently described symptom, often associated with dysphagia and fever [2-4]. It should be noted that the patient did not have any complaints related to dental pathology, which is why no evaluation of the dental pieces was carried out in primary health care or at the hospital level. However, during the physical examination, special attention should be given to the oral cavity, including dental conditions, trismus, purulent exudate, uvula deviation, bulging of the posterior pharyngeal wall, as well as lymph node enlargement, swelling, redness, or warmth in the cervical region and restriction on neck movement [4,5]. 

Clinical history and physical examination raise a strong suspicion of cervical abscess, which is extremely relevant in the context of primary health care, where there is limited immediate access to imaging exams. In these cases, referral to the emergency service is necessary to carry out additional diagnostic tests. The most commonly used imaging method is the CT scan, which shows thickening of the pharyngeal wall with fluid collection and sometimes signs compatible with the presence of gas [4]. In our patient, the image was consistent with an abscess, with a gas present inside due to abscess extension and secondary esophageal perforation, which can be one of the consequences of this condition.

Regarding treatment, broad-spectrum empirical antibiotic therapy is recommended to cover a wide range of gram-positive, gram-negative, and anaerobic bacteria, while awaiting culture isolation [1]. These infections often involve multiple microorganisms; isolates from Streptococcus viridans group, oral mucosa commensals, as well as Streptococcus pyogenes and Staphylococcus aureus, have been described [1,3-5]. Surgical treatment with abscess drainage can be an option, especially in patients with compromised airways or when the abscess size exceeds 3 cm [1,6]. In our patient's case, the abscess dimensions were 7 x 6 x 4cm, and when they explored the infection site orally, it was already spontaneously draining.

Early diagnosis of this condition is crucial, as delays in treatment initiation can lead to the progression of infection into the deep cervical spaces, resulting in serious complications such as mediastinitis, pericarditis, jugular vein thrombosis, sepsis, laryngeal edema, increased morbidity, and mortality [1,2]. This is a reason for all clinicians to be alert to this entity.

Given the main causes of retropharyngeal abscesses, patients should be made aware of the importance of maintaining good oral hygiene and treating any dental pathologies. Likewise, with advancing age, good oral health becomes essential in order to prevent the loss of dental parts that can compromise chewing. The awareness of oral health and the identification of problems concerning oral pathology may be one of the functions of the family doctor. 

Family Medicine is defined by the World Organization of Family Doctors (WONCA) as a person-centered medical discipline that integrates comprehensive, continuous, and community-based health care [7]. It is usually the first point of medical contact within the health system, providing open and unlimited access to users and dealing with all health problems regardless of age, gender, or any other characteristic of the person in question. 

## Conclusions

As reported throughout this clinical case, retropharyngeal abscess is a serious infection with high morbidity and mortality, and early diagnosis is essential to prevent complications. In this sense, family doctors can play a fundamental role in the early detection of this entity, since they are often the gateway to health services, and because of their knowledge of the patient as a "biopsychosocial" whole. 

The signs and symptoms present in a retropharyngeal abscess are, as we have seen, very non-specific and can easily go unnoticed by patients, so the family doctor can be a key player in the suspicion and early referral of these cases, allowing for timely treatment, with antibiotic therapy or surgical drainage, if necessary, reducing more serious complications, such as the risk of airway obstruction and systemic infection.
